# Glycemic Control after Sleeve Gastrectomy and Roux-En-Y Gastric Bypass in Obese Subjects with Type 2 Diabetes Mellitus

**DOI:** 10.1007/s11695-017-3061-3

**Published:** 2017-12-20

**Authors:** Ville Wallenius, Eveline Dirinck, Lars Fändriks, Almantas Maleckas, Carel W le Roux, Anders Thorell

**Affiliations:** 1 Department of Gastrosurgical Research and Education, Sahlgrenska Academy, University of Gothenburg, Gothenburg, Sweden; 2 Department of Endocrinology, Diabetology, and Metabolism, University of Antwerp, Antwerp, Belgium; 3 Department of Surgery, Medical Academy, Lithuanian University of Health Sciences, Kaunas, Lithuania; 4 Diabetes Complications Research Centre, Conway Institute, University College of Dublin, Dublin, Ireland; 5 Department of Clinical Science at Danderyd Hospital, Karolinska Institutet and Department of Surgery, Ersta Hospital, Stockholm, Sweden

**Keywords:** Obesity, Type 2 diabetes, Gastric bypass, Sleeve gastrectomy, Glycemic

## Abstract

**Background:**

Roux-en-Y gastric bypass (LRYGB) has weight-independent effects on glycemia in obese type 2 diabetic patients, whereas sleeve gastrectomy (LSG) is less well characterized. This study aims to compare early weight-independent and later weight-dependent glycemic effects of LRYGB and LSG.

**Methods:**

Eighteen LRYGB and 15 LSG patients were included in the study. Glucose, insulin, GLP-1, and GIP levels were monitored during a modified 30 g oral glucose tolerance test before surgery and 2 days, 3 weeks, and 12 months after surgery. Patients self-monitored glucose levels 2 weeks before and after surgery.

**Results:**

Postoperative fasting blood glucose decreased similarly in both groups (LRYGB vs. SG; baseline—8.1 ± 0.6 vs. 8.2 ± 0.4 mmol/l, 2 days—7.8 ± 0.5 vs. 7.4 ± 0.3 mmol/l, 3 weeks—6.6 ± 0.4 vs. 6.6 ± 0.3 mmol/l, respectively, *P* < 0.01 vs. baseline for both groups; 12 months—6.6 ± 0.4 vs. 5.9 ± 0.4, respectively, *P* < 0.05 for LRYGB and *P* < 0.001 for LSG vs. baseline, *P* = ns between the groups at all times). LSG, but not LRYGB, showed increased peak insulin levels 2 days postoperatively (mean ± SEM; LSG + 58 ± 14%, *P* < 0.01; LRYGB − 8 ± 17%, *P* = ns). GLP-1 levels increased similarly at 2 days, but were higher in LRYGB at 3 weeks (AUC; 7525 ± 1258 vs. 4779 ± 712 pmol × min, respectively, *P* < 0.05). GIP levels did not differ. Body mass index (BMI) decreased more after LRYGB than LSG (− 10.1 ± 0.9 vs. − 7.9 ± 0.5 kg/m^2^, respectively, *P* < 0.05).

**Conclusion:**

LRYGB and LSG show very similar effects on glycemic control, despite lower GLP-1 levels and inferior BMI decrease after LSG.

**Electronic supplementary material:**

The online version of this article (10.1007/s11695-017-3061-3) contains supplementary material, which is available to authorized users.

## Introduction

Obesity is a major risk factor for development of type 2 diabetes mellitus (T2DM)—and a key target in its treatment. Bariatric surgery, and in particular Roux-en-Y gastric bypass (LRYGB), induces marked and well-characterized effects on glycemic control that are superior to best medical treatment [1]. Bariatric surgery has therefore recently been recommended as part of the diabetes treatment, extending to patients with T2DM with body mass index (BMI) below 35 kg/m^2^ [2, 3]. The observed effects on glucose control are rapid and obviously in part non-weight dependent [4].

A non-weight-dependent mechanism after surgery involving bypass of the small intestine is a rapid and pronounced postprandial increase of incretin hormones, e.g., glucagon-like peptide (GLP)-1 [5, 6]. Despite its “simplistic” nature without intestinal bypass and despite incomplete elucidation of the underlying mechanisms, laparoscopic sleeve gastrectomy (LSG) seems to have good effects on glucose control as well, at least in the middle–long term. Some data indicate that gastric emptying is not delayed after LSG, suggesting that the operation does not exert its effects solely through “restriction.” [7–9] Supporting this idea is the observation in pregnant rat dams showing that they can increase food intake substantially even after LSG surgery [10]. Despite the lack of intestinal bypass, it seems that release of several incretins, such as GLP-1 and peptide YY (PYY), is increased in a similar way after LSG as after LRYGB, at least in non-diabetic patients [11]. A potential mechanism could be, in line with the situation after LRYGB, that, due to the reduced reservoir capacity of the stomach, undigested food enters the small intestine more rapidly, which in turn increases stimulation of the hormone-producing entero-endocrine cells of the intestinal mucosa.

The aim of this study was to compare and characterize, in detail, the changes in glycemic control in patients with T2DM and obesity who undergo LRYGB or LSG. In order to differentiate between weight- and non-weight-dependent effects, measurements were performed at various times after surgery.

## Methods

### Patients

Thirty-four subsequent patients with T2DM who fulfilled the inclusion criteria were asked to participate and were included from the waiting list for bariatric surgery at two sites in Sweden, the Sahlgrenska University Hospital, Gothenburg, and Ersta Hospital, Stockholm. The inclusion criteria were BMI 35–50 kg/m^2^, age 18–60 years, and T2DM requiring any available diabetes medications, but not only dietary regimen. Exclusion criteria were inability to understand the Swedish language or to adhere to study instructions. At Sahlgrenska Hospital, all patients were randomized to either method (seven LRYGB, five LSG). At Ersta Hospital (12 LRYGB, 10 LSG), the patients were designated for either LRYGB or LSG depending on the patients’ preference and the surgeons’ judgment, e.g., severe reflux disease was considered a contraindication for LSG. There was no systematic bias for the choice of one or the other method. Patients were asked to daily record their fasting (FBG) and 90 min postprandial glucose levels (PPBG; after individual breakfast), as well as all use of diabetes medications during 2 weeks before and 2 weeks after surgery. Postoperative adjustments of diabetes medications were handled by the physician usually in charge of the patient’s diabetes treatment. Weight, waist circumference, and height were recorded with patients wearing only underwear after an overnight fast. The usual 2-week preoperative low-energy diet (LED) was omitted in order not to influence preoperative or early postoperative glycemic control [12]. Eighteen patients underwent LRYGB and 15 underwent LSG. One patient who was not operated on by the intended technique at Sahlgrenska was excluded. One patient was lost to follow-up after the 3-week follow-up. The total follow-up rate was 94% (see Supplementary Fig. 1 for flow chart of inclusion). Data for GLP-1 and GIP were lost for four patients in the LSG group at 12 months; therefore, data are shown for 11 LSG patients in Figs. 6d and 7d. All patients were of Caucasian origin. The study was registered at ClinicalTrials.gov identifier NCT01984762.

### Study Ethics

This study was approved by the Regional Ethical Review Board of Gothenburg (study reference number 016-12), and the study was conducted according to the principles of the Helsinki declaration. All patients gave written informed consent.

### Modified Oral Glucose Tolerance Test (MOGTT)

MOGTT with 30 g of glucose (compared to standard OGTT 75 g) dissolved in 150 ml water was performed at four occasions: 3 weeks preoperatively and 2 days, 3 weeks, and 12 months postoperatively. The glucose dose was reduced in order to, as far as possible, avoid side effects such as vomiting and dumping in the postoperative phase. MOGTT was performed after a 12-h fast. Patients did not take any antidiabetic medications, and no long-, intermediate-, or fast-acting insulin was administered on the morning of the test. Glucose, insulin, glucagon-like peptide (GLP)-1, and gastric inhibitory peptide (GIP) levels were measured at 0, 15, 30, 60, 90, 120, 150, and 180 min after intake of the oral glucose dose. In case the glucose level declined to basal level at any time before 180 min, the blood sampling was terminated at that time.

### Surgery

All patients underwent a laparoscopic LRYGB or LSG by experienced bariatric surgeons. LRYGB was performed by a five-port technique with the use of a linear stapler for both the gastrojejunostomy and the jejuno-jejunostomy. A running suture was used for closure of the remaining defects as previously described [13]. The length of the Roux limb (alimentary limb) was typically 120 cm and the biliopancreatic limb 50 cm. For LSG, a linear stapler was used for resection of the stomach over a 35–36 F bougie from 3 to 5 cm proximal to the pylorus to 1–2 cm distal to the angle of His. Postoperative dietary recommendations included intake of fluids and semi-solid food for 2 weeks, and solid food thereafter. Patients were instructed to adhere to a diet rich in protein and with a calorie content of approximately 800–1000 kcal for the first six postoperative weeks.

### Glucose, Insulin, GLP-1, and GIP Measurements

At the Sahlgrenska University Hospital, blood glucose was measured using StatStrip according to the manufacturer’s instructions (Nova Biomedical, Waltham, MA, USA). At Ersta Hospital, blood glucose was measured using the YSI Model 2300 Stat Plus glucose analyzer according to the manufacturer’s instructions (Yellow Springs Instruments, OH, USA). Insulin was measured by an electrochemiluminescence immunoassay “ECLIA” using a Cobas e immunoassay analyzer according to the manufacturer’s instructions (Roche Diagnostics, Dublin, Ireland). The intra-assay variation of the insulin measurement was 1.1–1.4% (CV) and the inter-assay variation 3.5–3.7% (CV). The cross-reactivity with IGF-1 was 0.04%. GLP-1 and GIP were measured using ELISAs according to the manufacturer’s instructions (Merck Millipore, Solna, Sweden; Human total GIP ELISA, product number EZHGIP-54K, and Multi Species GLP-1 Total ELISA, product number EZGLP1T-36K). The intra-assay variation for GLP-1 was 1–2% (CV) and the inter-assay variation < 12% (CV). For GIP, the intra-assay variation was 3–8.8% (CV) and the inter-assay variation 1.8–6.1% (CV).

### Calculation of HOMA-IR and HOMA-B (%)

HOMA-IR, an index of insulin resistance, was calculated based on the morning fasting plasma insulin and blood glucose levels using the formula: HOMA-IR = (insulin (μIU/ml) × FBG (mmol/l))/ 22.5. HOMA-B (%) is a measure of pancreatic β-cell activity and was calculated using the formula: ((20 × insulin (μIU/ml))/(FBG (mmol/l)) – 3.5)% [14]. It should be noted that the assessment of the dynamics of beta cell functioning in the stability of a fasting state and using a single mathematical model such as HOMA-B is less reliable than the assessment of a relatively stable factor such as insulin resistance [15].

### Statistics

All statistics were performed using Prism 5 and 7 (version 5.0a and 7.0a). Logarithmic transformation was performed where indicated in order to obtain equal variances. Area under the curve for glucose, insulin, GLP-1, and GIP during the oral glucose tolerance tests was calculated using the trapezoidal rule. One-way ANOVA with Dunnett’s post hoc test was used for analyzing changes from baseline to the different postoperative times within groups. Paired and unpaired Student’s *t* test was used for single comparisons between baseline and postoperative values and between the groups, respectively. *χ* ^2^ test was used to compare numbers of patients on/off diabetes medications 12 months after surgery. A *P* value of < 0.05 was considered statistically significant.

## Results

### Anthropometry and Diabetes

There were no differences in age, gender, weight, BMI, diabetes duration, or basal HbA1c between the LRYGB and LSG groups of patients (Table 1). The reduction in body weight and BMI at 12 months postoperatively was significantly greater after LRYGB compared to LSG, whereas the improvement of HbA1c did not differ between groups (Table 1). The number of patients needing diabetes medications and/or insulin treatment decreased after surgery and was not different between the LRYGB and the LSG group, neither at baseline nor postoperatively up to 12 months (Table 2). The diabetes medication and insulin doses in the individual patients in the respective group at baseline and 12 months postoperatively are shown in Table 3.

**Table 1:** Anthropometric measures of the study groups at baseline and after surgery

			RYGB		SG		
			Mean ± SEM	*P*base^RYGB^	Mean ± SEM	*P*base^SG^	*P*groups
Gender	(F/M)		10:8		7:8		0.62
Age	(years)	Base	51.2 ± 1.6		51.9 ± 1.9		0.78
Body weight	(kg)	Base	112.9 ± 3.6		109.0 ± 3.4		0.43
		12 mo	84.3 ± 3.0	< 0.001	85.2 ± 3.0	< 0.001	0.83
Δ Body weight	(kg)	12 mo	29.5 ± 2.6		22.1 ± 1.2		< 0.05
BMI	(kg/m^2^)	Base	38.6 ± 0.8		36.9 ± 0.7		0.14
		12 mo	28.8 ± 0.7	< 0.001	28.6 ± 0.6	< 0.001	0.86
Δ BMI	(kg/m^2^)	12 mo	10.1 ± 0.9		7.9 ± 0.5		< 0.05
EWL	(%)	12 mo	73.0 ± 5.0	< 0.001	69.1 ± 4.4	< 0.001	0.57
Waist/hip ratio		Base	1.00 ± 0.02		1.02 ± 0.02		0.58
		12 mo	0.96 ± 0.02	< 0.01	0.94 ± 0.02	< 0.001	0.53
Diab duration	(years)	Base	5.7 ± 0.6		6.5 ± 1.1		0.54
HbA1c	(%)	Base	61.8 ± 3.9		55.7 ± 2.1		0.20
		6 w	50.3 ± 2.9	< 0.001	48.7 ± 2.0	< 0.001	0.66
		12 mo	40.5 ± 2.0	< 0.001	43.9 ± 2.7	< 0.01	0.34
Δ HbA1c	(%)	0–12 mo	− 15.9 ± 5.2	< 0.001	− 11.5 ± 2.6	< 0.001	0.47

**Table 2:** Total numbers of patients and types of medications before and 12 months after surgery

	T2DM treatment	OAD	Insulin	OAD + insulin	All treatm	No treatm
		*n* (%)	*n* (%)	*n* (%)	*n* (%)	*n* (%)
RYGB	Before	9 (53)	1 (6)	7 (41)	17 (100)	–
	After	6 (35)	1 (6)	0 (0)	7 (41)	10 (59)
SG	Before	11 (73)	0 (0)	4 (27)	15 (100)	–
	After	5 (33)	0 (0)	1 (7)	6 (40)	9 (60)

Total no. of patients on/off medications 12 months after surgery were compared by ***χ*** ^2^ test

**Table 3:** Diabetes medications in all subjects before and 12 months after surgery

	Subject number	Diabetes treatment, baseline			Diabetes treatment, 12 months		
		Metformin (mg)	OAD (mg)	Insulin (IU)	Metformin (mg)	OAD (mg)	Insulin (IU)
RYGB	1	1500			0		
	2			168			0
	3	1500			0		
	4	1000			0		
	5	1000	Glimepiride (1000), liraglutide (1.8)		0	0	
	6	2000	Liraglutide (1.2)	40	0	0	0
	8	1000			0		
	9	1000			0		
	10	2000			0		
	11	2000		28	0		0
	14	1500			0		
	16	500		95	–		–
	19	3000		94	500		0
	20	2550	Glibenklamide (1.75)		1000	0	
	21	1000			0		
	24	1000		wn	3000		0
	26	2000		25	500		0
	34	1500		30	1000		0
SG	12	1500		46	0		0
	13	3000	Glimepiride (4)		0	0	
	17	2000	Glimepiride (4)		2000	Glimepiride (2)	
	18	3000	Glimepiride (2)		1000	0	
	22	1500			0		
	23	1000–1500			0		
	25	1500	Pioglitazone (30)		1000	0	
	27	3000		80	3000		28
	28	1500	Glibenklamide (7)		2000	Glibenklamide (3.5)	
	29	1000			0		
	30	500			0		
	31	1500			0		
	32	2550		58–70	0		0
	33	2000		80	1000		0
	39	500			0		

Liraglutide was administered by subcutaneous injections

*wn* when needed

### Fasting Glucose, Insulin, and HOMA

**Fig. 1:**
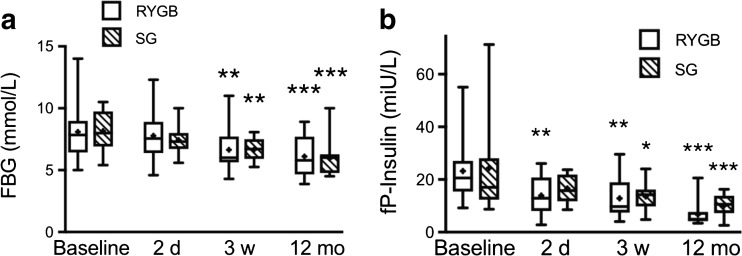
**a** Fasting blood glucose (FBG) and **b** fasting plasma insulin (fP-Insulin) levels at baseline, 2 days, 3 weeks, and 12 months after LRYGB or LSG surgery. The box plots show the median and 95% confidence intervals for AUCs. The plus sign shows the mean. *P < 0.05, **P < 0.01, and ***P < 0.001 for ANOVA with Dunnett’s post hoc test for comparisons within groups at the different times after surgery vs. baseline

Fasting blood glucose (FBG) concentrations were not significantly changed in either group at 2 days after surgery, but were decreased in both groups at 3 weeks and 12 months after surgery (Fig. 1a). Fasting plasma insulin was decreased as early as on day 2 in both groups; however, only significantly in the LRYGB patients at this time (Fig. 1b). At 3 weeks and 12 months after surgery, fasting insulin was similarly decreased in both groups. HOMA-IR was significantly improved compared to baseline from day 2 to 12 months after surgery within both LRYGB and LSG groups, and did not differ between the groups at baseline, or at any time after surgery (Table 4). HOMA-B (%) improved both after SG and RYGB, but the difference reached significance only in RYGB compared to baseline at 2 days and 12 months, and there were no significant differences between the groups at any time (Table 4).

**Table 4:** HOMA-IR and HOMA-B (%) at baseline and 2 days, 3 weeks, and 12 months after surgery

	RYGB	SG	Paired-samples *t* test		Independent-samples *t* test
			RYGB	SG	
	Mean ± SD	Mean ± SD	*P* value	*P* value	*P* value
HOMA-IR					
Baseline	9.2 ± 6.4	8.4 ± 6.2	–	–	0.63
2 days	4.5 ± 2.4	5.4 ± 1.8	< 0.001	< 0.05	0.13
3 weeks	3.8 ± 2.0	4.0 ± 1.6	< 0.001	< 0.001	0.52
12 months	1.9 ± 1.2	2.3 ± 1.1	< 0.001	< 0.001	0.31
HOMA-B (%)					
Baseline	151 ± 132	130 ± 116	–	–	0.63
2 days	87 ± 87	94 ± 46	<0.05	0.387	0.25
3 weeks	107 ± 81	93 ± 40	0.141	0.344	0.99
12 months	56 ± 42	87 ± 56	0.001	0.245	0.11

All statistical analyses were performed with logarithmically transformed values in order to obtain normal distribution. Paired-samples *t* test was performed comparing baseline values with values at 2 days, 3 weeks, and 12 months, respectively. Independent-samples *t* test was performed between RYGB and SG

### MOGTT

**Fig. 2:**
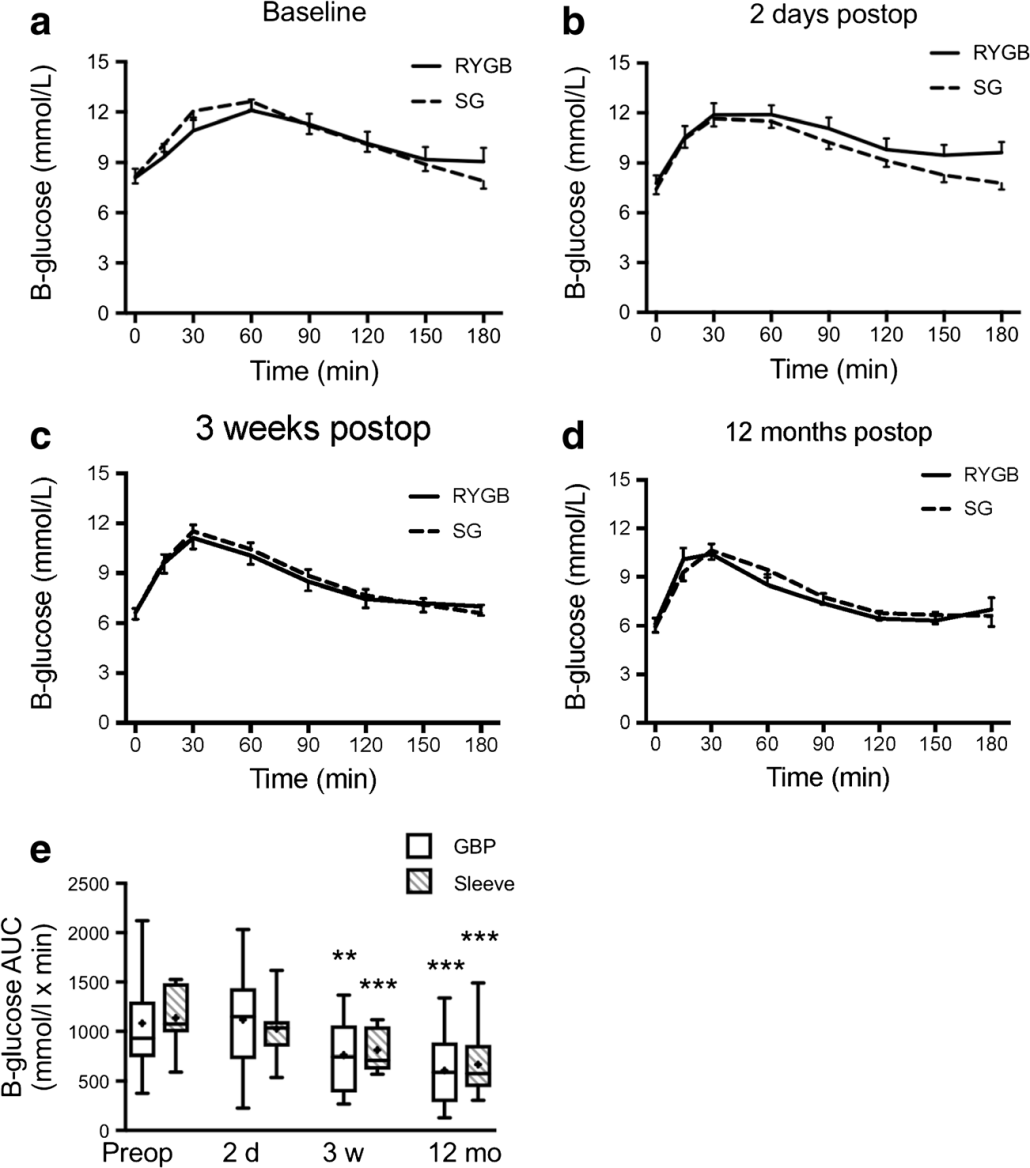
Blood glucose levels (B-glucose) 0–180 min after a modified 30 g oral glucose tolerance test at **a** baseline, **b** 2 days, **c** 3 weeks, and **d** 12 months after LRYGB or LSG. Graphs (**a**)–(**d**) show means ± SEM. **e** The box plots show the median and 95% confidence intervals for AUCs for the corresponding times. The plus sign shows the mean. **P < 0.01 and ***P < 0.001 for ANOVA with Dunnett’s post hoc test for comparisons within groups at the different times after surgery vs. baseline

**Fig. 3:**
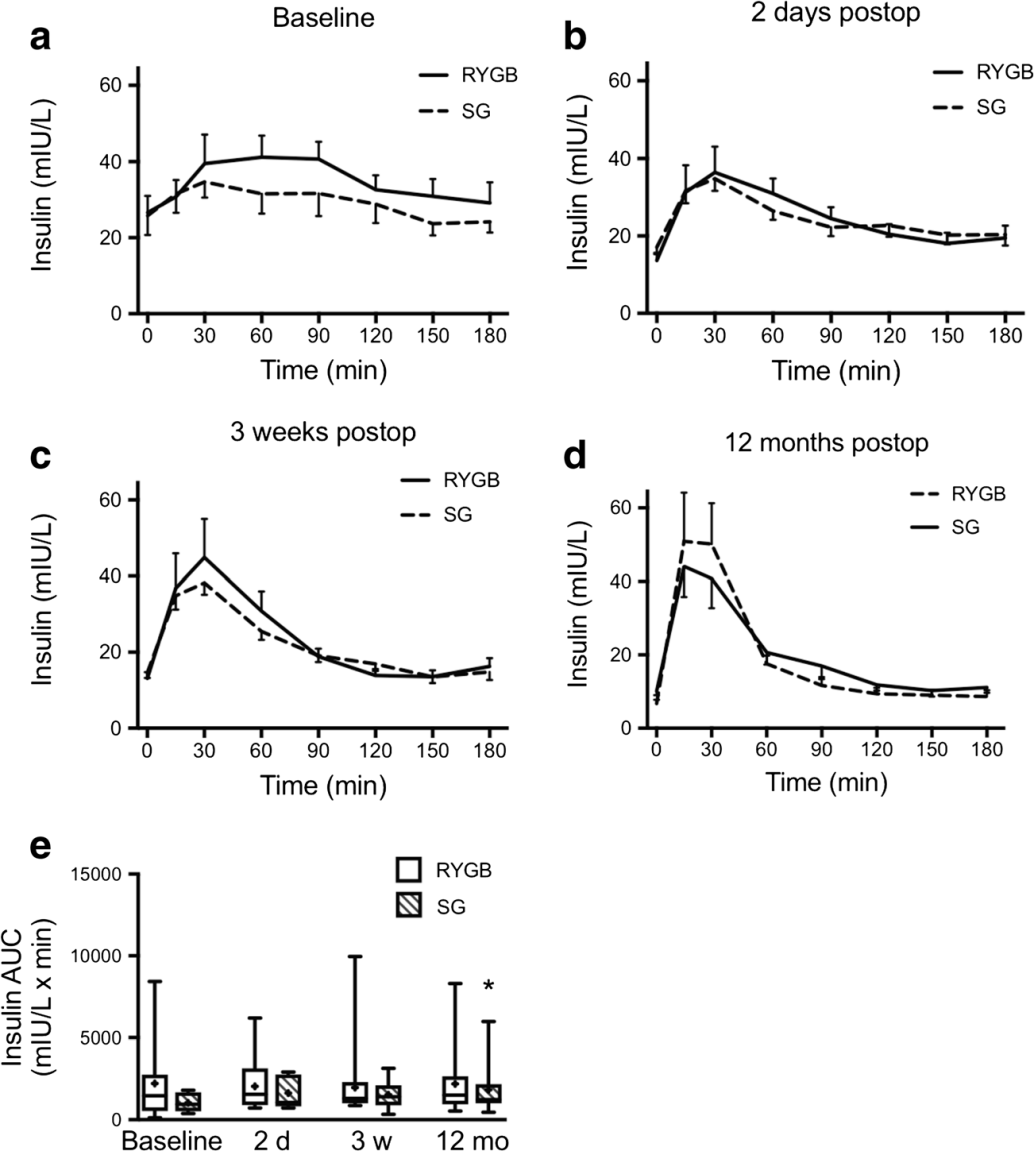
Plasma insulin levels 0–180 min after a modified 30 g oral glucose tolerance test at **a** baseline, **b** 2 days, **c** 3 weeks, and **d** 12 months after LRYGB or LSG. Graphs (**a**)–(**d**) show means ± SEM. **e** Box plots show the median and 95% confidence intervals for AUCs for the corresponding times. The plus sign shows the mean. *P < 0.05 for ANOVA followed by Dunnett’s post hoc test for comparisons within groups at the different times after surgery vs. baseline

**Fig. 4:**
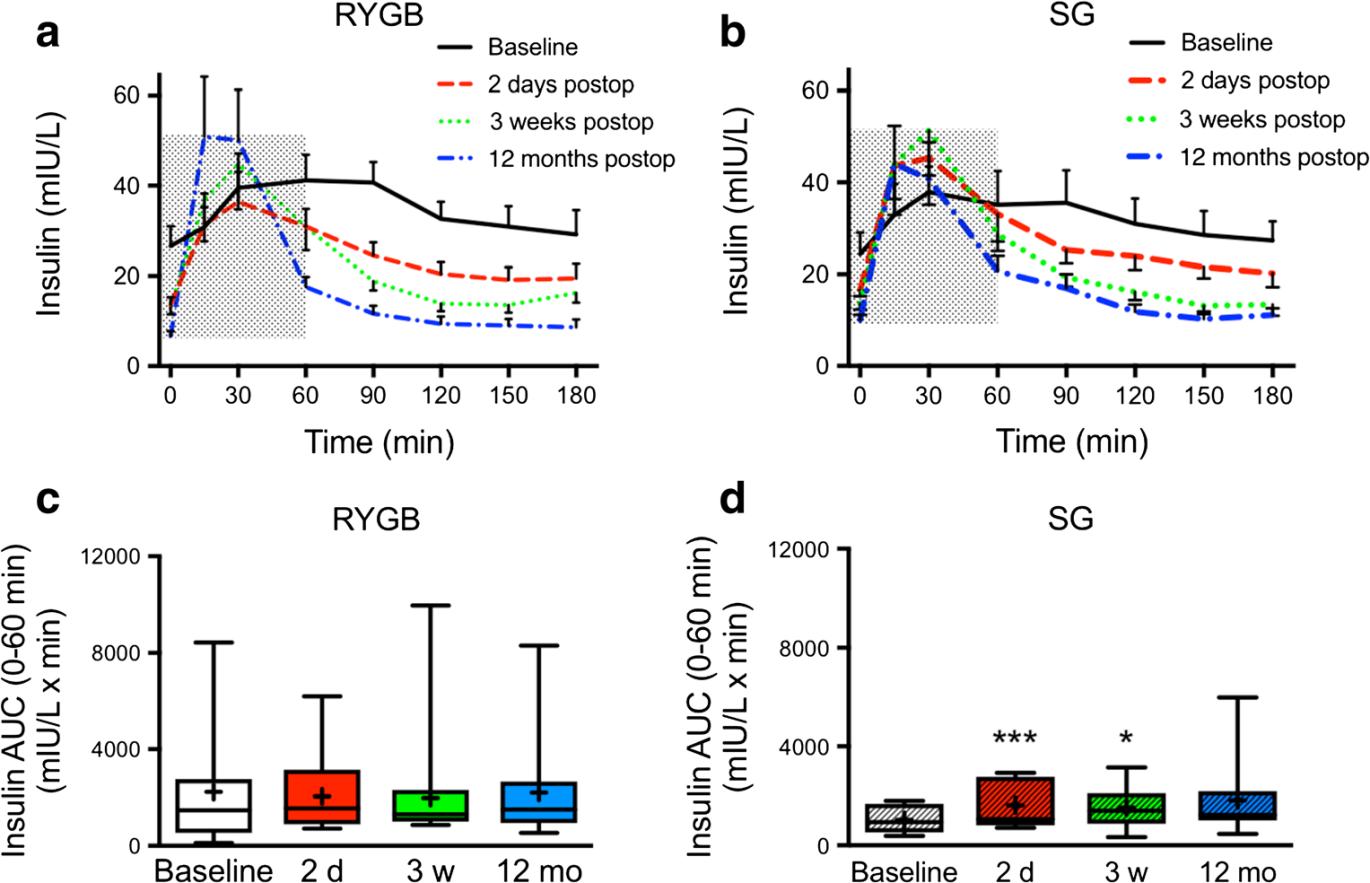
Within-group plasma insulin levels 0–180 min after a modified 30 g oral glucose tolerance test at baseline, 2 days, 3 weeks, and 12 months after **a** LRYGB or **b** LSG. Graphs (**a**) and (**b**) show means ± SEM. Box plots show the median and 95% confidence intervals for AUCs 0–60 min (gray hatched areas in **a** and **b**) for **c** LRYGB and **d** LSG. The plus sign shows the mean. AUCs *P < 0.05 and ***P < 0.001 for ANOVA followed by Dunnett’s post hoc test for comparisons within groups at the different times after surgery vs. baseline

In the LSG group, 3 out of 15 patients were able to ingest only part of the glucose dose 2 days after surgery because of nausea (20 g in two patients and 15 g in one patient). All 2-day calculations were repeated with exclusion of these three patients, but that did not change the outcomes in any significant way. Glucose and insulin levels during MOGTT in RYGB and SG from baseline to 12 months after surgery are shown in Figs. 2 and 3. Glucose clearance was significantly improved in both groups at 3 weeks after surgery as calculated using AUC 0–180 min and continued to improve in both groups until 12 months after surgery (Fig. 2e). The preoperative insulin release in response to the glucose load was blunt and showed a prolonged peak from 15 to 90 min in both groups (Fig. 3a). After surgery, already from day 2 and on, the insulin responses were more rapid and distinct and the peak occurred at 15–30 min in both groups (Fig. 3b–d). Total AUC for insulin was not different between the groups and only significantly decreased in the LSG group at 12 months compared to baseline (0–180 min; Fig. 3e). However, the seemingly elevated insulin peak area at 2 days in LSG encouraged us to analyze insulin peak AUCs 0–60 min. This revealed a clearly significant increase in the LSG, but not in the LRYGB group, both at day 2 and at 3 weeks postoperatively (Fig. 4c, d).

### Patient Self-Measured Glucose Levels Pre- and Postoperatively

**Fig. 5:**
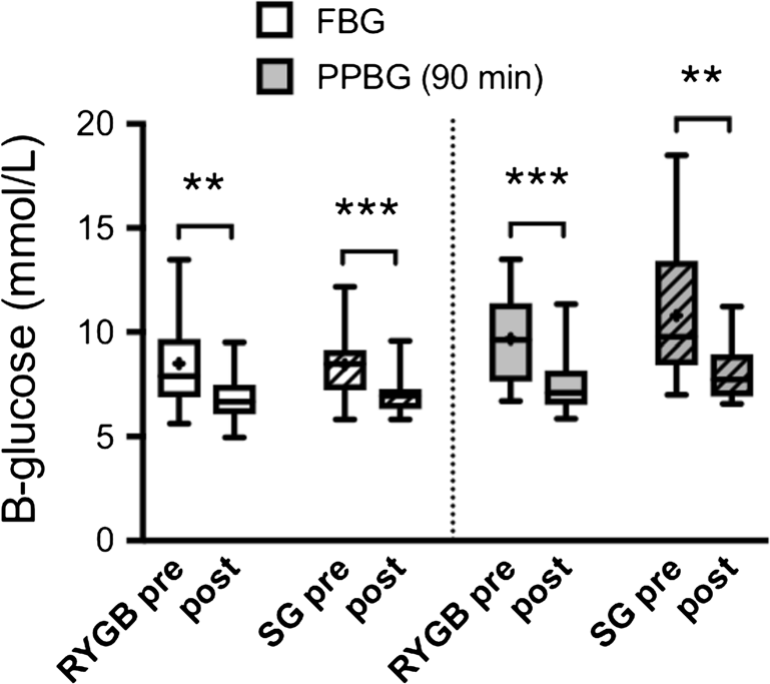
Patient self-reported morning FBG (left 4 box plots) and 90 min PPBG (right 4 box plots) measured during 14 days preoperatively (pre) and 14 days postoperatively (post) after LRYGB or LSG, showing the median and 95% confidence intervals. The plus sign shows the mean. The patients were not put on LED before surgery to avoid effects of fasting on glucose. White bars represent LRYGB and hatched bars represent LSG. **P < 0.01 and ***P < 0.001 for paired *t* test within groups before and after surgery

Figure 5 shows patient self-measured FBG and 90 min PPBG after an individual breakfast during the first 2 weeks before compared to 2 weeks immediately after surgery. In line with the data from the MOGTTs, these self-reported glucose measurements clearly showed significantly decreased FBG and 90 min PPBG levels in both groups of patients with no differences between the groups (Fig. 5).

### Glucagon-Like Peptide (GLP-)1 Levels after Surgery

**Fig. 6:**
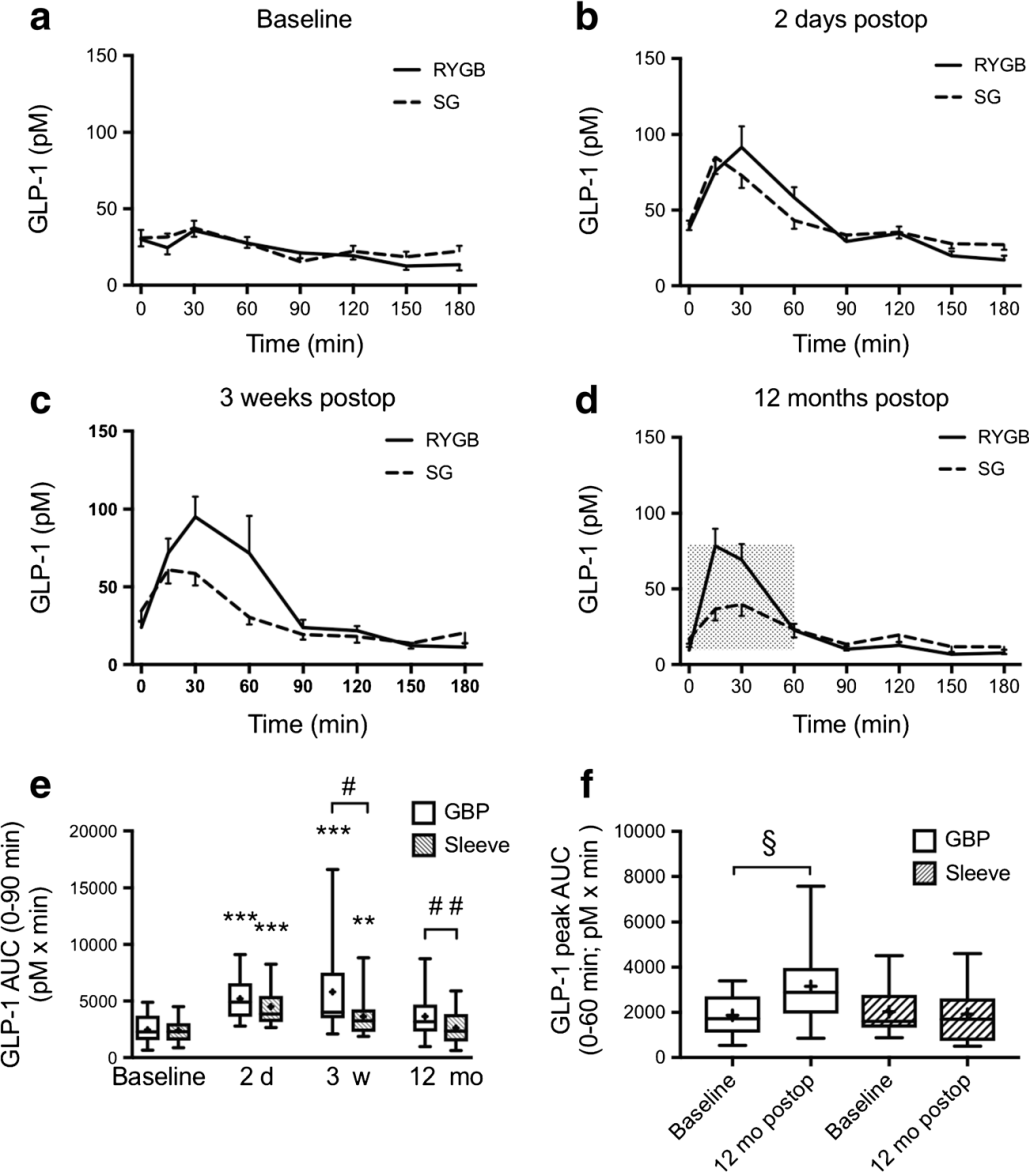
Plasma GLP-1 levels 0–180 min after a modified 30 g oral glucose tolerance test at **a** baseline, **b** 2 days, **c** 3 weeks, and **d** 12 months after LRYGB or LSG. Graphs (**a**)–(**d**) show mean ± SEM. **e** Box plots showing median and 95% confidence intervals for AUCs for GLP-1 at corresponding times. The plus sign shows the mean. **f** Peak AUC for GLP-1 (0–60 min) after LRYGB and LSG. **P < 0.01 and ***P < 0.001 for ANOVA followed by Dunnett’s post hoc test for comparisons within groups at the different times after surgery vs. baseline. ^#^ *P* < 0.01 and ^##^ *P* < 0.001 for unpaired *t* test between LRYGB and LSG. ^§^ *P* < 0.05 paired-samples *t* test within group. *N* = 18 for LRYGB and 15 for LSG in all panels, except panel (**d**) where *n* = 11 for LSG

The GLP-1 levels during MOGTT were substantially increased 2 days after both LRYGB and LSG with no differences in AUCs between the groups (Fig. 6a, b, e).

Three weeks after surgery, GLP-1 levels, in response to the MOGTT, were maintained in the LRYGB patients, whereas they had started to decline in the LSG patients and declined further at 12 months to levels not different compared with baseline (Fig. 6c, d). Although the total AUC at 12 months was not increased compared to baseline in LRYGB, it was still significantly higher in the LRYGB group compared to LSG group (Fig. 6e). Analysis of *peak* AUC (0–60 min; hatched area in Fig. 6d) showed an increase in the GLP-1 peak after LRYGB also at 12 months compared to baseline, whereas there was no increase after LSG (Fig. 6f).

### Gastric Inhibitory Peptide (GIP) Levels after Surgery

**Fig. 7:**
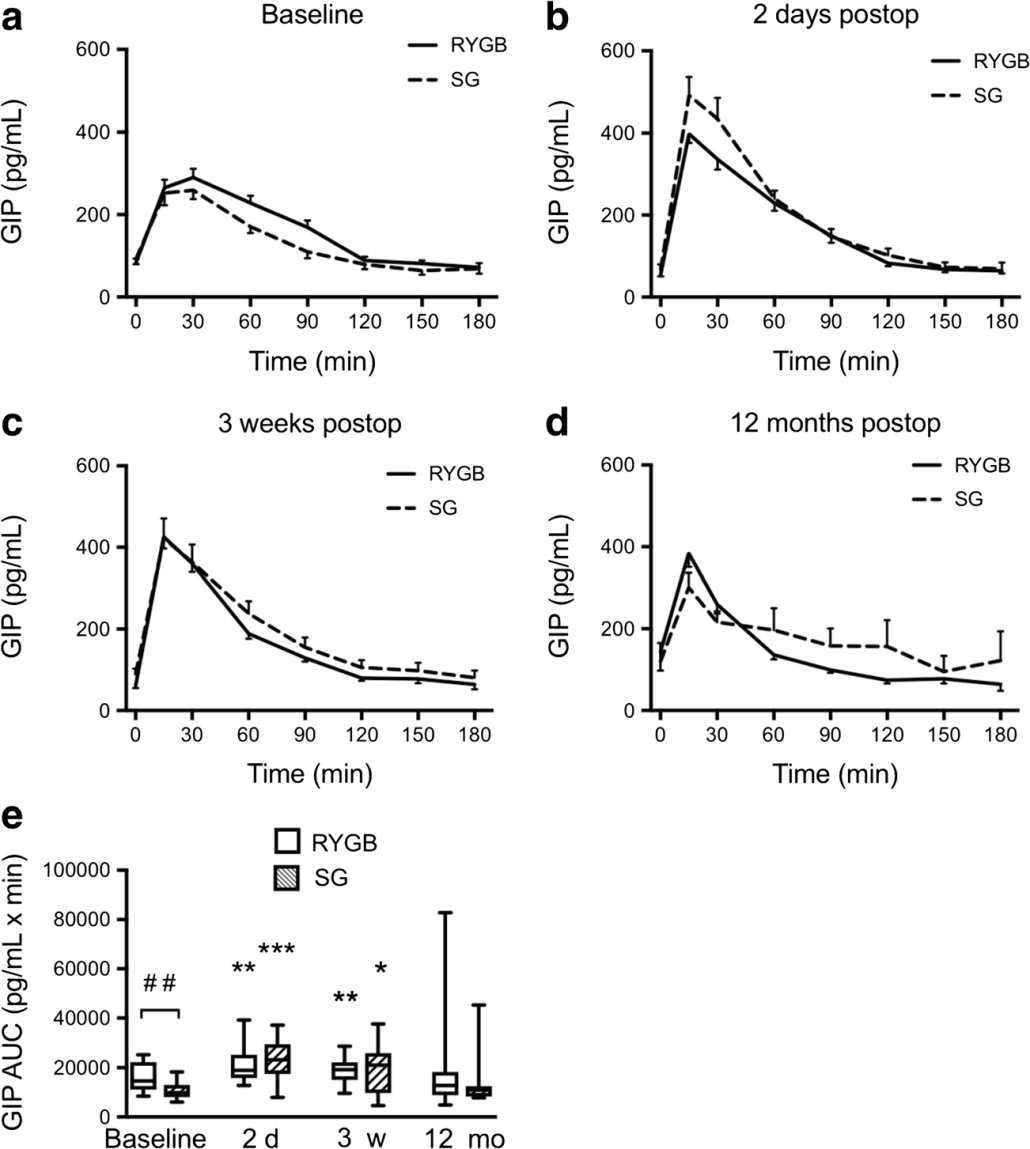
Plasma GIP levels 0–180 min after a modified 30 g oral glucose tolerance test at **a** baseline, **b** 2 days, **c** 3 weeks, and **d** 12 months after LRYGB or LSG. Graphs (**a**)–(**d**) show means ± SEM. **e** Box plots showing median and 95% confidence intervals for AUCs for GIP at corresponding times. The plus sign shows the mean. *P < 0.05, **P < 0.01, and ***P < 0.001 for one-way ANOVA followed by Dunnett’s post hoc test. ^##^ *P* < 0.001 for between-groups *t* test LRYGB vs. LSG. *N* = 18 for LRYGB and 15 for LSG in all panels, except panel (**d**) where *n* = 11 for LSG

GIP levels in response to MOGTT were significantly increased in both LSG and LRYGB patients at 2 days and 3 weeks after surgery compared to baseline (Fig. 7a–c). They then started to decline and were not significantly increased at 12 months in either group compared to baseline (Fig. 7d, e). AUC GIP was higher in LRYGB compared with LSG at baseline, but no differences were noted between groups postoperatively (Fig. 7e).

## Discussion

Sleeve gastrectomy (LSG) is currently one of the most common bariatric operations worldwide, although the long-term effects on body weight and diabetes have not yet been characterized in detail. A number of studies focusing on long-term effects are ongoing [16–19], but very few data on the very early weight-independent effects of LSG have been presented in patients with obesity and T2DM. Therefore, we performed a comparative study between LSG and LRYGB, to examine the early postoperative effects on glycemic control, supplemented by the effects after 1 year.

The most important finding of the present study in patients with T2DM was that the early effect of SG on glucose control and insulin secretion was equal compared to that of RYGB. Although based on a relatively limited number of patients, we found that RYGB elicited a statistically significant increase in the GLP-1 response at 3 weeks. However, this did not translate into a greater improvement of glucose metabolism variables in the RYGB group at that moment compared to the LSG group. This is well in line with experimental data from animal studies, showing that even complete absence of GLP-1 does not change the effect of LSG on weight decrease and glucose metabolism [20]. In relation to LRYGB, it has been speculated that decreased release of a hitherto uncharacterized anti-incretin or “decretin” factor from the bypassed foregut, which would suppress insulin release, could be of importance [21]. If this factor is of importance after LSG as well, it would most probably be released from the area along the major curvature of the stomach, which is removed, rather than from the duodenum since this is not bypassed in LSG. Moreover, this would also fit with the rapid recurrence of diabetes in RYGB patients or diabetic rats where food is reintroduced to the remnant stomach via a gastrostomy cannula [22, 23]. On the other hand, many other factors have been suggested to influence the metabolic response after bariatric surgery, e.g., bile acids and changes of the microbiota [24]. Our study did not focus on the latter aspects, and as such, a contribution of these parameters cannot be excluded nor proven. Moreover, calorie restriction is known to be a potent insulin sensitizer [25]. The improvement in glucose metabolism, observed in both groups, could be influenced by the preoperative fasting and postoperatively decreased caloric intake. The authors assume caloric intake was similarly decreased within the first days and weeks after SG and RYGB despite the fact that the two procedures are anatomically completely distinct.

In corroboration with previous studies [17, 18, 26, 27], the weight loss was statistically significantly lower after LSG compared to LRYGB 1 year after surgery. Despite this and despite the subtle differences in the insulin and GLP-1 curves, mentioned above, these differences did not translate into differences in clinically relevant diabetes measures or diabetes medication use between the groups at any time during the first postoperative year. The fact that both groups lost more than 20% of their initial body weight after 1 year corresponds to the observed improvement in glucose metabolism. These improvements can be equally important when the weight loss has been achieved by non-dietary measures, as shown in the Look AHEAD study [28].

Postoperative vomiting and nausea (PONV) is more commonly reported after LSG than after LRYGB. Accordingly, three of the LSG patients were not able to ingest the entire 30 g glucose dose at the first 2-day MOGTT. This did, however, not influence the data presented in any significant way since the results were the same irrespective if these patients were included or not. On post-op day 2, HOMA-IR was significantly reduced in both groups, whereas fP-insulin and HOMA-B (%) were significantly reduced only in the LRYGB patients. On the other hand, the peak AUC 0–60 min for insulin was increased only in the LSG patients at this time. Although we cannot exclude that the lack of differences between groups was due to a type II error, these findings might suggest that the anti-diabetes effects of these two operations are distinct, with a somewhat more pronounced effect by LRYGB on insulin sensitivity, and by LSG on β-cell activity.

The measurements at 1 year postoperatively were performed in order to evaluate the combined effect of weight-dependent and non-weight-dependent mechanisms. There were no significant differences in glycemic control between LSG and LRYGB at 1 year, as assessed by HbA1c levels, MOGTT measurements, the patients self-reported FBG and PPBG, or the numbers of patients off anti-diabetes medications. The exception being HOMA-B (%), that still was significantly decreased after LRYGB but not LSG. The effect of LSG, with removal of a major part of the stomach, could be related to the decreased reservoir capacity and the transfer of undigested food to the small intestine where the release of hindgut factors, e.g., GLP-1 and GIP, is stimulated and could exert beneficial effects on insulin and glucagon release. The levels of incretin hormones, in response to a somewhat higher oral glucose load, were previously shown to be relatively similar in *non-diabetic* RYGB and SG patients [11]. In the present study, however, the GLP-1 stimulatory effect of LSG was transient and started to subside already 3 weeks after surgery. Whether this reflects a true difference in GLP-1 response after LSG compared to LRYGB in patients with or without diabetes is not entirely clear. An alternative explanation is that the differences in GLP-1 response are associated with the use of a lower energy load in our study (30 g glucose vs. a liquid test meal containing 15 g carbohydrates, 25 g protein, and 28 g fat).

Limitations of this study include the relatively small numbers of patients, which obviously is associated with a risk that we were unable to statistically demonstrate some additional true differences between the groups. Moreover, all patients were not randomized. Anthropometric data were not different in any aspect between the groups at baseline. To assess glucose metabolism, ingestion of a low-dose glucose drink was used. This choice was made to avoid vomiting and dumping, despite the fact that this low-dose drink has not been extensively validated. A similar load of glucose has, however, previously been shown to induce a satisfactory response in intestinal hormones after bariatric surgery [29]. Moreover, using a glucose drink as opposed to a mixed meal test limits the interpretability as human data indicate proteins are also able to elicit a dose-dependent incretin effect [30]. Detailed food diaries, in particular during the first 3 weeks after surgery, were not collected. Therefore, the authors cannot be certain that patients consumed the recommended post-op diet. Recent studies suggest that differences in glycemic control between the groups may appear with longer follow-up time [19].

Strengths of this study were the careful monitoring of glucose metabolism at several times both very early and up to a year after surgery by MOGTT, and the “real-life” self-monitoring data from patients during 2 weeks before and 2 weeks after surgery on glucose levels at fasting and postprandially after breakfast.

In conclusion, in this study, LSG exerted early beneficial effects on glycemia that were nearly indistinguishable from those after LRYGB. GLP-1 secretion in response to an oral glucose load was only transiently increased after LSG, in contrast to LRYGB where GLP-1 peak levels were still increased at 1 year postoperatively. The BMI loss after 1 year was lower after LSG than LRYGB. Elucidation of other mechanisms that contribute to the positive glycemic effects of LSG may hold the key to understanding how patients can be helped to maintain long-term glycemic control after bariatric surgery.

## Conclusion

LRYGB and LSG induced similar effects on glycemic control, both very early after surgery and up to 1 year after surgery, despite lower GLP-1 levels and inferior decrease of BMI after LSG.

## Electronic supplementary material

Supplementary Figure 1 Flow chart of the assessment and selection of patients for the study. (GIF 46 kb)

High resolution (TIFF 191 kb)
