# Supplementary figures and images for: Glycemic Control after Sleeve Gastrectomy and Roux-En-Y Gastric Bypass in Obese Subjects with Type 2 Diabetes Mellitus
Source: Obes Surg. 2017 Dec 20;28(6):1461–72. doi: 10.1007/s11695-017-3061-3 (PMC5973990; doi:10.1007/s11695-017-3061-3)

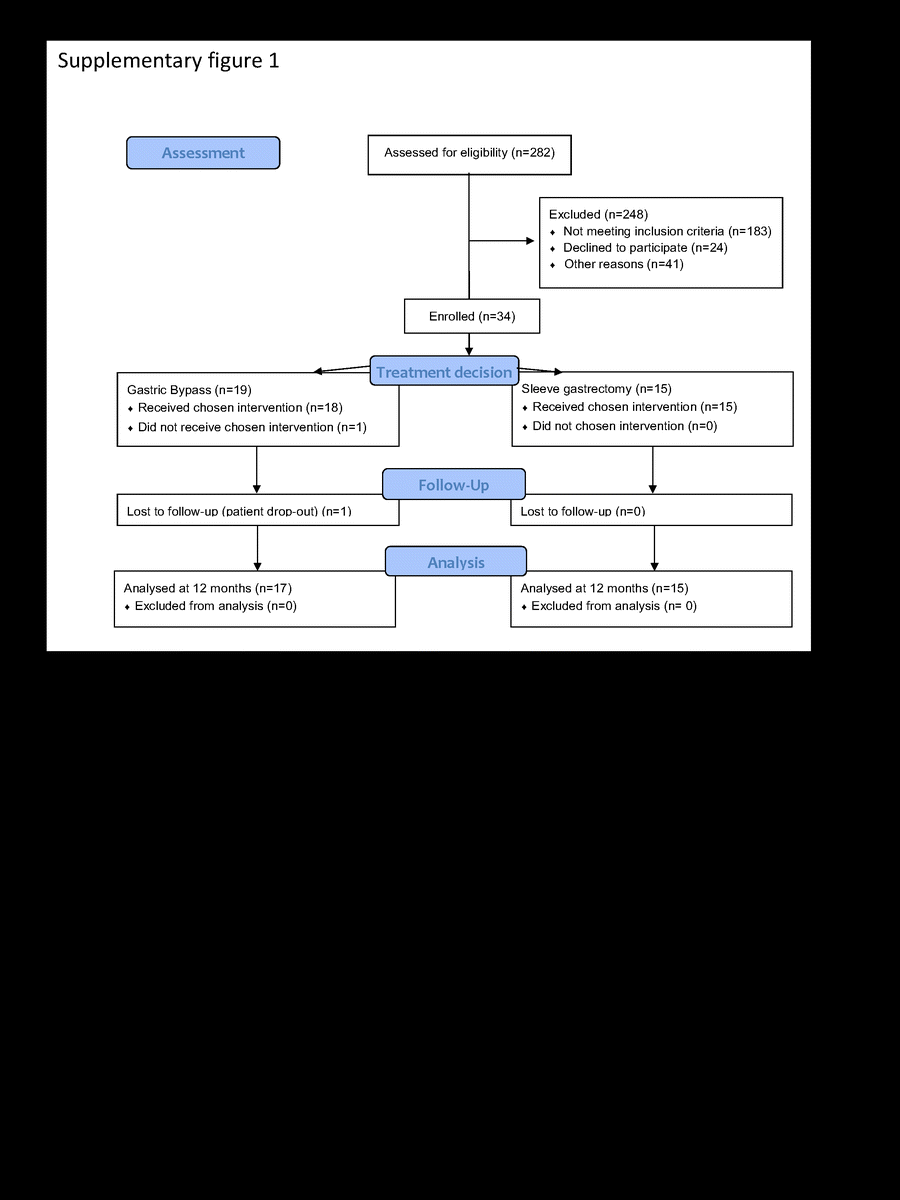

Supplement: Supplementary file 1 — Flow chart of the assessment and selection of patients for the study. (GIF 46 kb) [file 11695_2017_3061_FIG8_ESM.gif]

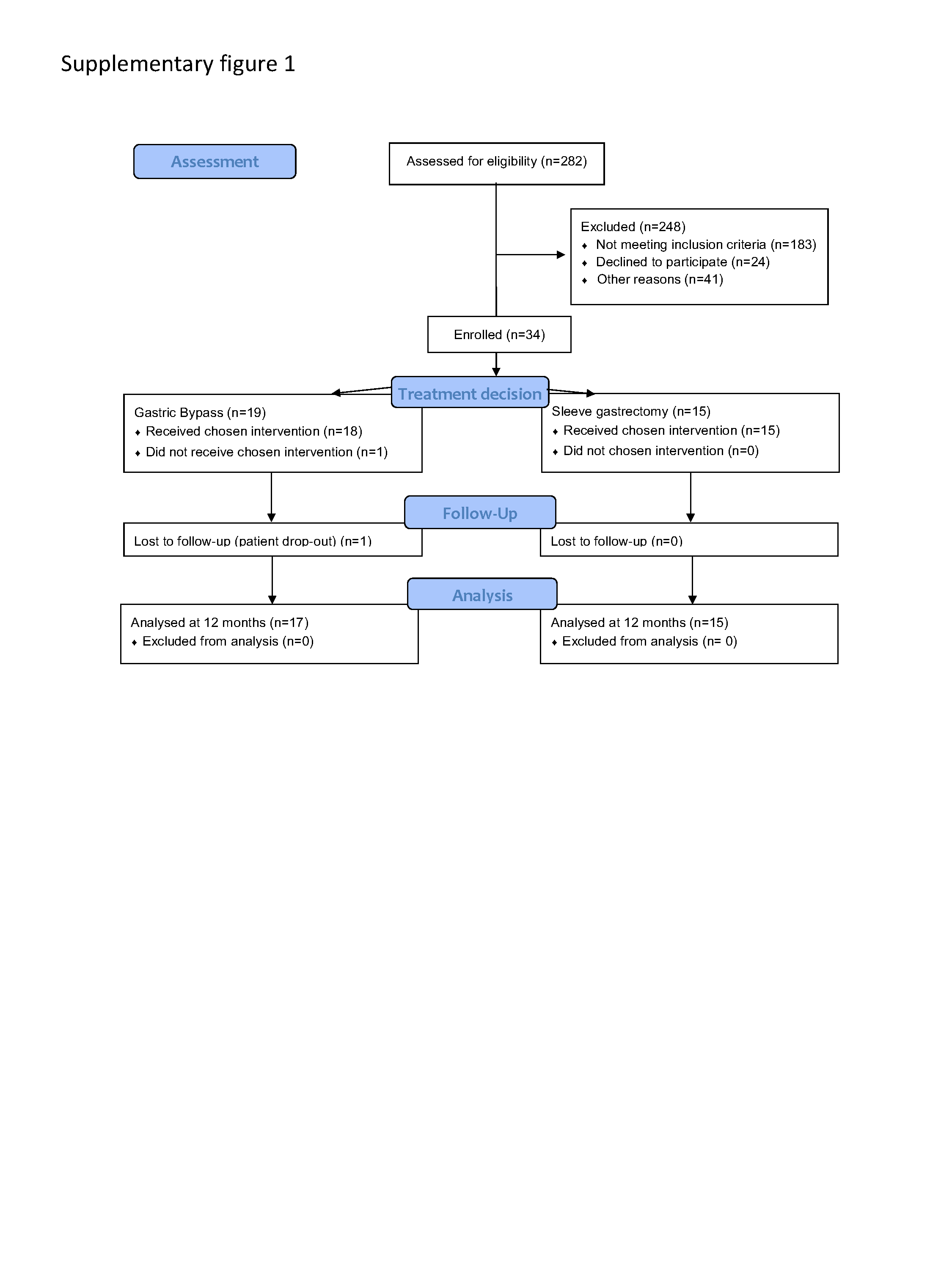

Supplement: Supplementary file 2 — High resolution (TIFF 191 kb) [file 11695_2017_3061_MOESM1_ESM.tiff]
